# The Connection between Immunocompetence and Reproduction in Wildlife

**DOI:** 10.3390/life13030785

**Published:** 2023-03-14

**Authors:** Matthias Bernhard Stope

**Affiliations:** Department of Gynaecology and Gynaecological Oncology, University Hospital Bonn, 53127 Bonn, Germany; matthias.stope@ukbonn.de; Tel.: +49-228-287-11361

**Keywords:** veterinary research, veterinary medicine, evolution, immunology, pregnancy

## Abstract

Reproduction rate is important for the survival of animal populations. During gravidity, a trade-off occurs between the individual well-being of gravid females and investment in offspring. Due to the high synthesis and energy requirements for the growing fetus, other physiological activities are downregulated in pregnant females. This causes changes in the composition of the reproductive microbiome and a decreased immune response to presented antigens and pathogens. As a result, the immunocompetence of gravid wild animals declines. In general, therefore, increased infection rates during pregnancy can be observed in all wildlife species studied. In the course of evolution, however, this has apparently evolved as a suitable strategy to ensure the survival of the population as a whole.

## 1. Introduction

The immune system protects the organism from invading agents (e.g., toxins, viruses, bacteria, parasites) and pathologically altered endogenous structures (e.g., malignant cells, pathological proteins). For a successful immune response, the two systems of rapid innate and specific adaptive immunity are activated. Immunocompetence refers to the ability of an organism to respond to an antigenic stimulus. The opposite is immunodeficiency. Immunocompetence concerns both the cellular response of immune cells and the response of the organism as a whole. In vertebrates, the main function of the immune system is to protect against infectious agents and largely determines the physiological fitness of the animal. In most cases, changes in population size are based on the ability of individuals to respond adequately to changes in environmental factors, including pathogens. In this context, increased mortality mainly affects pregnant individuals and individuals of neonatal age. Therefore, not only individual survival, but also the survival and size of the population are determined by the immunocompetence of individuals [1,2]. This is accompanied by the fact that immunocompetence is largely genetically determined and thus has undergone evolutionary progression. However, the extent of immunocompetence can also be modulated by internal (e.g., food, disease) and external factors (e.g., stress, season) [3,4]. Therefore, the regulation of immunological responses also depends on the available resources. Immunocompetence, like all other evolutionarily optimized mechanisms, is also subject to the pressure of a biological economy. The maximal immune response is not necessary in every case. Rather, the response must be adapted to the specific (infection) status and physiological constitution of the animal [2].

## 2. The Concept of Immunocompetence and Reproduction in Animals

When considering immunocompetence and reproduction in animals, a clear line must be drawn between wild animals and domesticated farm animals. Whereas in wildlife physiological performance has been adapted to evolutionary benefit, in farm animals the physiological cost–benefit calculation has given way to anthropogenic requirements. Significant differences in immune response can occur even in closely related animal species. For example, even closely related pig varieties exhibit varying levels of immunocompetence. This can be exploited in crossbreeding to obtain more robust livestock. It appears that wild and only slightly domesticated animal species are generally more immunocompetent than modern livestock races [5,6,7]. This is possibly due to the fact that domesticated animals, as well as humans, are no longer subjected to the rules and mechanisms of evolution.

Nevertheless, the issue of immunocompetence in reproduction has been studied much more intensively in animal breeding than in wildlife biology. Initially, livestock were selected for specific production traits that optimize yield. However, general resilience properties have been lost as a result. Many species used for livestock production became significantly more susceptible to disease, especially to infections during gravidity. In livestock production, attempts are now being made to evaluate and consider physical robustness, including immunocompetence [8,9]. In the case of infections, these approaches also go beyond animal health and welfare. Bacterial infectious diseases are particularly problematic, for example, in poultry farming. On the one hand, microbial contamination in meat constitutes a risk for the consumer. On the other hand, the current situation leads to the heavy use of antibiotics. This, in turn, enhances the formation of resistant microorganisms and leads to the antibiotic contamination of meat-based foods [10]. The immunocompetence of livestock is linked to the quality of the food produced. Therefore, among other things, the immunological characteristics of the animals are measured and optimized during breeding.

For these reasons, the present review on the correlation of immunocompetence and reproduction in animals was based exclusively on data from wild animals. This may be perceived as a limitation, but the immunological behavior of domesticated animals in the reproductive phase corresponds only to a limited extent to the natural state evolved through evolution.

## 3. The Interplay of Physiology, Immunology, and Reproduction in Wildlife

In the field of wildlife biology and medicine, little is known about immunological dynamics in wild animals. Most of the data are from laboratory animals, obtained under low-pathogen and well-defined environmental conditions. In contrast, in the wild, wildlife is exposed to a much broader range of antigens. In addition, differences between individuals and populations of the same species are larger than in genetically uniform laboratory or farm animals, making statistically evaluable observations challenging. Varying individual immunocompetence appears to be of great importance to the fitness and survival of a wildlife population [11,12]. Biologically, immune response efficacy is particularly relevant in reproduction. If the immunocompetence of gravid females is impaired, this may lead to the termination of the gravidity and thus to a lower reproductive rate [13,14].

Multiple vertebrate lineages have developed different reproductive strategies during evolution. For example, in mammals alone, there are numerous differences in the ontogenetic origin and organization of the maternal–fetal interface [15,16,17]. These distinct processes are accompanied by different expression profiles of the genes involved [18]. During gravidity in vertebrates, significant physiological and metabolic alterations occur. This extends beyond the reproductive tract to other tissues and organs, such as muscle tissue, adipose tissue, liver, and the gastrointestinal tract. These endocrine-controlled processes tend to distribute nutrients throughout the maternal organism and provide glucose and amino acids, in particular, to the growing fetus [19,20]. Therefore, reproduction, above all in mammals, is one of the most energy-consuming processes in the female life cycle. Gravidity and the subsequent phase of lactation are characterized by enormous synthesis efforts [21,22]. The high metabolic burden, together with environmental factors (e.g., food availability, temperature), leads to a conflict between self-maintenance and reproduction. This trade-off between individual life expectancy and reproductive success affects many areas of physiology, including individual immunocompetence [23,24].

## 4. The Maternal–Fetal Interface in Live-Bearing Vertebrates

All live-bearing vertebrates form a membrane system for the fetus, the egg membranes (amnion, chorion, decidua). These protect the fetus and serve the exchange of substances with the maternal organism. In the contact zone of maternal and fetal tissue, the vessel systems of mother and fetus are in contact for physiological exchange. This area is defined as the placenta and is the attachment or fusion of the fetal membranes to the uterine mucosa. It has evolved in all classes of vertebrates except birds [25].

**Figure 1:**
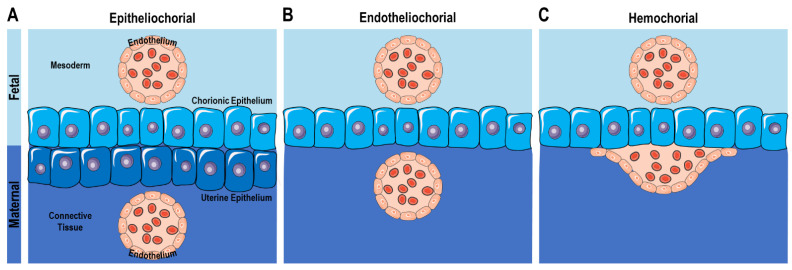
The maternal–fetal interface of mammalian placentas defined by the structure of the tissue barrier between maternal circulation and fetus. Epitheliochorial (**A**) The maternal blood system is segregated from the chorionic epithelium by endothelium and uterine epithelium (e.g., porcine, bovine). Endotheliochorial (**B**) The maternal blood system is segregated from the chorion only by the endothelium of the blood vessel (e.g., dogs, cats). Hemochorial (**C**) Maternal blood has direct contact with the chorionic epithelium (e.g., humans, rodents).

With the exception of the platypus (*Ornithorhynchus anatinus*) and recent species of echidna (*Tachyglossus aculeatus, Zaglossus* sp.), a placenta is formed for reproduction in all mammals. During gravidity, the mammalian placenta takes over almost all the functions of the fetal organs, which are not yet functional, and enables the exchange of substances in both directions. Usually, the numerous slightly different types of placentation in mammals are divided into three simplified types [26,27]. In epitheliochorial placentation, fetal chorionic epithelium and maternal uterine epithelium are adjacent to each other, separating the two vessel systems (Figure 1A). In endotheliochorial placentation, the maternal epithelial layer is missing, and fetal trophoblast cells directly contact the maternal blood vessels (Figure 1B). Finally, in the hemochorial type, fetal trophoblast cells infiltrate the maternal tissue, destroy parts of the maternal vascular endothelium, and thus establish direct contact with the uterine arteries (Figure 1C).

The histological cellular organization of the maternal–fetal interface also determines molecular transport across the placenta. In rodents, significant levels of immunoglobulin are transferred to the fetal vessel system even before birth. In contrast, in species from the carnivore group, some passive immunity is transferred to the fetus only toward the end of gravidity. However, there are also animals, such as horses and pigs, in which the placental transfer of maternal antibodies to the fetus does not occur. In these species, maternal antibodies are transferred through breast milk only after birth [28,29].

The different placental types have evolved several times during vertebrate evolution, some independently of each other, and differ in structure and degree of maternal provisioning to the fetus. In live-bearing fish, trophonemata (finger-shaped projections of the uterine mucosa) placenta and yolk sac placenta are the most common histological placental types. In some fish species, the fetus remains in the follicle throughout embryonic development, in which placental structures form (follicular placenta). Furthermore, yolk sac and eating littermates or unfertilized eggs may also contribute to fetal provisioning [30,31]. Live-bearing amphibians and reptiles usually have much simpler placental structures. The fetus feeds on the contents of the egg, and adaptations of the female reproductive tract allow for the additional exchange of gases and nutrients between the fetus and mother. In most reptiles, placentation resembles epitheliochorial organization. In some cases, the amphibian fetus grows up in specialized body structures outside the reproductive tract, for example, in pouches on the back [32,33].

## 5. The Interaction of Reproductive Microbiome and Immunocompetence

Organisms of the reproductive microbiome significantly determine the development and activity of mammalian reproduction. This occurs through a direct interaction of the microorganisms with the maternal reproductive tract, for example, by inducing and maintaining a suitable local milieu. Furthermore, indirect factors may also contribute to successful reproduction. Microbial communities in the animal organism can modulate physiological properties and general reproductive signals, such as secreted scents or body surface coloration. Furthermore, the maternal microbiome is also transmitted to the offspring via skin, milk, and vaginal contact, which influences the health and resilience of the young [34]. Often the impact of the reproductive microbiome only becomes apparent when pathological changes occur during gravidity. The immune system, however, must first learn to tolerate the presence of commensal microorganisms [35,36]. Reproductive microbiome and immunocompetence influence each other. Thus, changes in microbial functionality in the reproductive tract also impact immunocompetence during gravidity. This is a very indirect characterization of immunomodulatory processes during gravidity but may also play a significant role.

Modern sequencing technologies enable high-throughput, culture-independent identification of microorganisms. Nevertheless, even for the human reproductive microbiome, the data situation is still limited. This is even more true for the male reproductive microbiome. The major problem is the low abundance of reproductive microorganisms and sampling without contamination of the sample with microorganisms from other areas of the genital tract. Moreover, the qualitative and quantitative ratios of the microbial community undergo changes during the female cycle. In the field of veterinary science, few data exist on the reproductive microbiome during gravidity. These largely concern farm and breeding animals [37,38,39]. In the field of wildlife biology and medicine, reliable data are even rarer. Some studies on the composition of the vaginal and uterine microbiome in giant pandas (*Ailuropoda melanoleuca*) and nonhuman primates suggest an association with gravidity outcome [40,41,42,43]. The microbiomes here revealed no correlation with species or phylogenetic relationships. Taking data from all animals, including domesticated species, a picture is emerging that despite commensal–host coevolution, closely related species can have very different microbiome compositions. On the other hand, species that are evolutionarily distant from each other can also show great similarities in their microbial composition [44]. The impact of the reproductive microbiome on the immunocompetence of gravid wild animals still has rather the status of a hypothesis, but cannot be dismissed out of hand.

## 6. The Detection of Molecular Markers of Immunocompetence during Gravidity

Another way to monitor immunocompetence is to measure immunologically relevant factors. Meanwhile, such immune markers have also been investigated in biology and veterinary research, and some suitable test methods are available [45]. Specific molecular markers, such as the transcription factor and modulator of Th2 immunity GATA3 or the glycolytic enzyme α-enolase, can be detected [46,47]. However, quantifying these molecular factors is methodologically complex. Since it is difficult to implement in the context of wildlife biology studies, many studies on wildlife immunology are limited to the analysis of antibody levels and their dynamics [45,48]. Furthermore, reactive oxygen species (ROS) have been identified as important regulators in reproductive processes. During gravidity, maternal and fetal oxygen production leads to the appearance of ROS and thus oxidative stress. In this context, ROS control several reproductive processes, from oocyte maturation to embryo implantation, and also have effects on immune system activity [49,50,51]. The available data on molecular factors during wild animal gravidity is obviously not comparable to the situation in human pregnancy. Nevertheless, some conclusions about the reproductive immunology of wildlife can be derived from the available studies.

Gravid Siberian hamsters (*Phodopus sungorus*), zebra finches (*Taeniopygia guttata*), and guinea pigs (*Cavia aperea*) demonstrated lower antibody responses after the administration of a test antigen compared to non-gravid animals [52,53,54]. In guinea pigs (*Cavia porcellus*), hemagglutinin and hemolysis tests also exhibited diminished complement system activity [52].

ROS are mainly considered in the context of inflammatory processes during gravidity [55]. Recent veterinary studies, however, suggest that ROS may also control the activity of immune cells in the reproductive tract [56,57]. In domesticated animals (Taurus cattle (*Bos taurus*), water buffalo (*Bubalus bubalis*)), the late phase of gravidity correlates with increased ROS levels and impaired host immune function [58,59,60]. In consequence, reproductive oxidative stress is also associated with increased susceptibility to pathogens [49,56]. No data are available for the field of wildlife biology and medicine on the ROS-dependent interference with reproductive immunocompetence. However, it can be assumed that the findings from domesticated animals are also applicable to wildlife.

## 7. Infections and Parasite Infestations during Gravidity

A cumulative parameter of immune system performance is susceptibility to infection and parasite infestation. In the context of reproduction, this also means that the reproductive rate of the individual as well as the population can be affected. A disease of the gravid animal endangers the animal itself, but also the unborn offspring. Furthermore, the immunological protection of the fetus may also be compromised, resulting in the neonatal young being at risk after birth. Most studies on immunocompetence during gravidity in wild animals are available on infection and parasite status.

Some studies with wild birds are available on this topic. In breeding great tits (*Parus major*), pathogens of the genus *Haematozoa* and *Plasmodium* [61,62] were increasingly detected; in flycatchers (*Ficedula hypleuca*) mainly *Trypanosoma* and *Haemoproteus* [63] were observed. In female willow ptarmigan (*Lagopus lagopus*) [64] and blue tit (*Cyanistes caeruleus*) [65], parasites have been shown to depress reproductive rates. Female falcons (*Falco sparverius*) have higher IgG serum levels during gravidity due to infection [66]. A meta-regression approach to correlate lowered immunocompetence during avian gravidity confirmed the above study data [67]. In purple martins (*Progne subis*), however, it was shown that infection with one pathogen had no effect on the gravidity of females. Only the coinfection with an additional pathogen resulted in a reduced reproductive rate [68,69]. Overall, in wild animals, resistance and viability, including reproduction, appears to decrease with increasing numbers of different parasites (polyparasitism) [68,70,71,72].

Studies of mammals demonstrated that in wild rabbits (*Oryctolagus cuniculus*) [71] and wood bison (*Bison athabascae*) [73] infections caused lower reproductive rates. In wild sheep (*Ovis canadensis*), nematodes and the corresponding antibodies were detected more frequently during gravidity and lactation [74,75].

In wild birds, susceptibility to pathogens often increases with increasing brood size [61,62,63]. This led to the hypothesis that available energy is also a limiting factor for reproductive immunocompetence. In birds, mice, and monkeys deprived of energy by physical stress, immunocompetence decreased. As during gravidity, animals were more frequently infected with viral pathogens [54,76,77]. Infections themselves already lead to higher metabolic costs in wild animals [78,79,80] and would consequently negatively affect gravidity.

## 8. Conclusions

**Figure 2:**
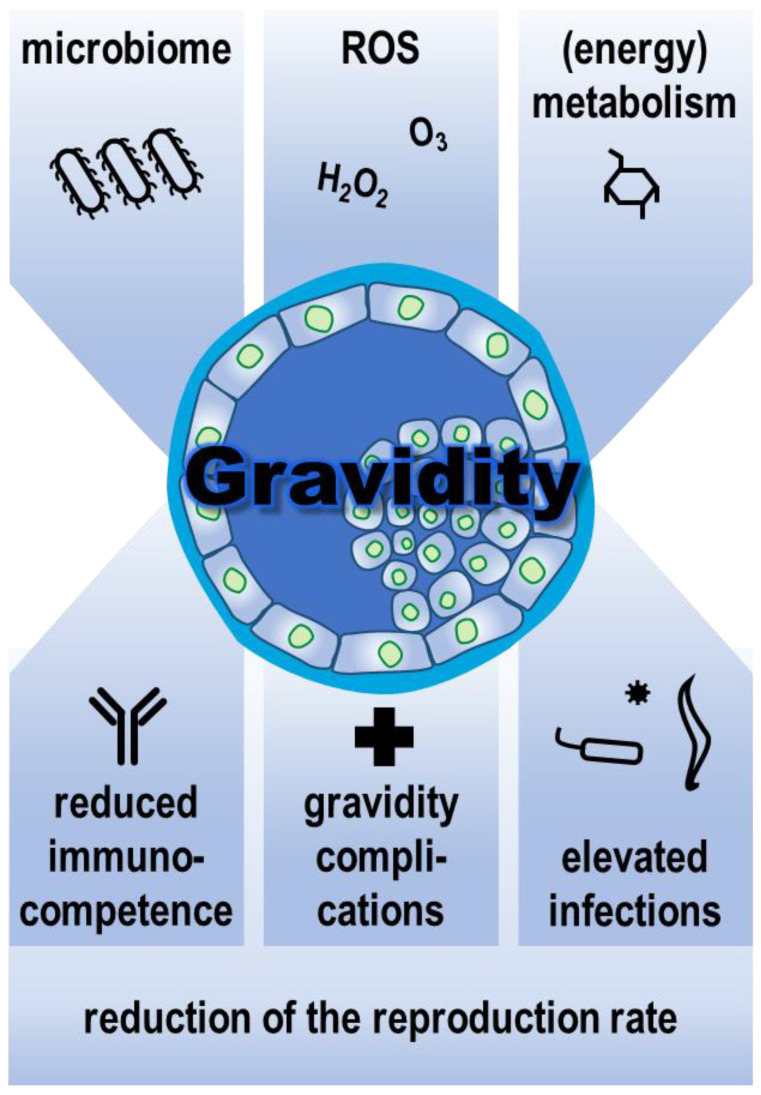
Alterations in microbiome quality, reactive oxygen species (ROS) formation, and metabolic activity affect physiology during gravidity. The resulting suppressed immunocompetence can lead to gravidity complications and increased infections, which ultimately lowers the reproductive rate.

In wildlife biology and medicine, access to physiological and pathological data is severely limited compared to livestock. Nevertheless, a certain picture can be drawn from the available studies on the modulation of immunocompetence in gravid wild animals (Figure 2).

A review of published studies shows a diminished capacity for immune response in gravid compared to non-gravid females of wild animals. As a consequence, there is evidence of a higher susceptibility to infections. These include viral and bacterial pathogens as well as parasites. One of the causes of decreased immunocompetence during gravidity is generally thought to be limited energy availability. With a nearly constant energy metabolism, gravid females must invest a sometimes considerable proportion of energy in the care and growth of the fetus. This is at the expense of other maternal physiological processes. This, along with other factors, may reduce the efficacy of the immune response. Reduced immunocompetence during gravidity appears to be a result of the trade-off between self-maintenance and reproductive rate. In different vertebrate classes, this strategy has evolved to varying degrees during evolution.

## Data Availability

Not applicable.
